# Myopericarditis associated with acute Zika virus infection: a case report

**DOI:** 10.1186/s12879-022-07454-8

**Published:** 2022-05-31

**Authors:** Camila Helena Aguiar Bôtto-Menezes, Izabella Picinin Safe, Ana Cláudia da Cunha Ferreira, Katia do Nascimento Couceiro, Armando Menezes Neto, Rafael Freitas Oliveira Franca, Guilherme Amaral Calvet, Ana Maria Bispo de Filippis, Edna Oliveira Kara, Marcia da Costa Castilho, Michele Souza Bastos, Carlos Alexandre Antunes de Brito, Kayvon Modjarrad, Nathalie Jeanne Nicole Broutet, Patrícia Brasil, Ludhmila Abrahão Hajjar, Marcus Vinícius Guimarães de Lacerda, ZIKABRA Study Team

**Affiliations:** 1 Tropical Medicine Foundation Dr. Heitor Vieira Dourado, Manaus, 69040-000 Brazil; 2 Department of Medicine, School of Health Sciences, University of Amazonas State, Manaus, 69065-001 Brazil; 3 Getúlio Vargas University Hospital, Federal University of Amazonas, Manaus, 69020-170 Brazil; 4 Institute Aggeu Magalhães, Oswaldo Cruz Foundation, Recife, 50740-465 Brazil; 5 Acute Febrile Illnesses Laboratory, Evandro Chagas National Institute of Infectious Diseases, Oswaldo Cruz Foundation, Rio de Janeiro, 21040-360 Brazil; 6 Flavivirus Laboratory, Oswaldo Cruz Institute, Oswaldo Cruz Foundation, Rio de Janeiro, 21040-360 Brazil; 7 Department of Sexual and Reproductive Health and Research, World Health Organization, 1211 Geneva, Switzerland; 8 Walter Reed Army Institute of Research, Silver Spring, 20910 USA; 9 Heart Institute, Medical School, University of São Paulo, São Paulo, 05403-900 Brazil; 10 Laboratory of Territory, Environment, Health and Sustainability, Leônidas and Maria Deane Institute, Oswaldo Cruz Foundation, Manaus, 69057-07 Brazil

**Keywords:** Zika, Myopericarditis, Myopericarditis virus-induced, Heart, Cardiac

## Abstract

**Background:**

Zika virus infection is commonly described as a mild and self-limiting illness. However, cardiac complications were associated with acute Zika virus infection.

**Case presentation:**

A 46-year-old woman without previous comorbidities with a 1-day history of symptoms tested positive for ZIKV by real time reverse transcriptase polymerase chain reaction (rRT-PCR). She was admitted two days after with clinical worsening, cardiac enzymes elevated, and cardiac imaging findings, and the diagnosis of myopericarditis was made. The patient was treated and presented significant clinical improvement after one year.

**Conclusions:**

Cardiac complication following ZIKV infection appears to be infrequent. Here, we report a rare case of viral myopericarditis caused by ZIKV infection.

## Background

Zika virus (ZIKV) is an arthropod-borne virus transmitted by *Aedes* sp. mosquitoes and has been reportedly detected in humans since 1954 [1]. However, following rapid spread around the globe and reports of neurological complications, in February 2016 the World Health Organization declared it a public health emergency of international concern [2]. Zika virus is regarded as causing a benign and self-limiting infection with symptoms lasting from few days to a week [3]. Occasionally, ZIKV infection has been associated with significant neurological complications, especially microcephaly and other congenital abnormalities, Guillain-Barré syndrome, encephalitis, myelitis, and meningoencephalitis, and other possible threats of the virus may have been overlooked [4]. Although the heart is amongst the organs reported to be affected by this infection, there are few studies describing the cardiac alterations associated to ZIKV infection [5–11]. Here, we describe cardiovascular complications occurred during the acute phase of ZIKV infection.

## Case presentation

**Fig. 1:**
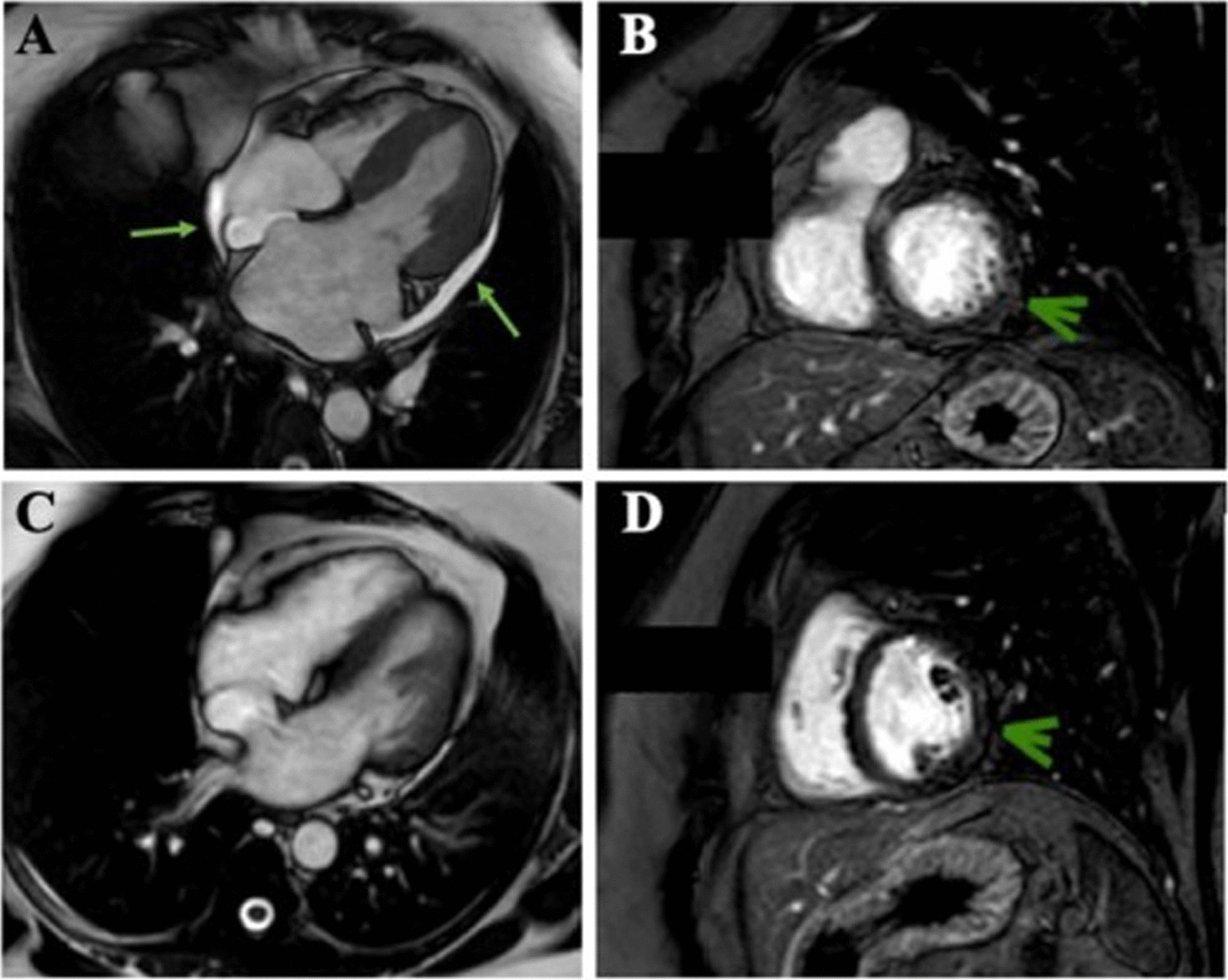
**A** cMRI of the heart in the FIESTA sequence in the four-chamber plane demonstrating pericardial effusion (green arrows). **B** cMRI of the heart in the FIESTA sequence in the short-axis demonstrating slight change in myocardial signal intensity (green arrow). **C** cMRI of the heart in the FIESTA sequence in the four-chamber plane, 1-year evolutionary control, demonstrating significant improvements in pericardial effusion. **D** cMRI of the heart in the FIESTA sequence in the short-axis, 1-year evolutionary control, demonstrating slight change in myocardial signal intensity (green arrow)

A 46-year-old healthy woman presented at the outpatient clinic of *Tropical Medicine Foundation Doctor Heitor Vieira Dourado* (FMT-HVD) with a one-day history of itchy maculopapular rash, fever, conjunctival hyperemia and periarticular edema preceded by myalgia and diarrhea. She lived in Manaus, Amazonas, Brazil and had no history of travelling within the previous month. Initial examination revealed a body temperature of 39.2 °C, bilateral conjunctivitis and maculopapular skin rash in trunk and limbs. Blood pressure (114/80 mm Hg) was normal and heart rate was 122 bpm. Blood and urine samples tested positive for ZIKV by real time reverse transcriptase polymerase chain reaction (rRT-PCR). Blood cell count was normal. After two days of the diagnosis, she returned with worsening of clinical presentation, fatigue, vomiting, diarrhea, dyspnea and lower limbs edema. Physical examination revealed hypotension (103/81 mm Hg) and persistence of the tachycardia (120 bpm). She was admitted to the ward and laboratory results showed an increase in the creatine kinase (CK) 4.027 U/L (normal < 170 U/L), creatine kinase-muscle/brain (CK-MB) 99U/L (normal < 25 U/L) and creatine kinase-muscle mass (CKMB) 29.4 U/L (normal < 4.3 U/L). The troponin T [0.27 ng/mL (normal < 0.04 ng/mL)], and myoglobin [> 500 ng/mL (normal < 107 ng/mL)] were elevated. The chest x-ray revealed hypotransparency on bases and enlarged cardiac silhouette. Her electrocardiogram (ECG) showed normal sinus rhythm. The 24-h ECG monitoring was normal. The echocardiogram (echo) revealed a left ventricular ejection fraction of 64% and a moderate pericardial effusion, without signs of cardiac tamponade. The cardiac magnetic resonance imaging (cMRI) revealed a thickening of the pericardium, moderate pericardial effusion (Fig. 1A) and an intramyocardial area with gadolinium enhancement involving the basal medial segment of the anterior septal wall (Fig. 1B) compatible with inflammatory changes observed in viral myocarditis based on Lake-Louis consensus criteria. The biventricular systolic function was preserved, and the myocardial perfusion was within the normal range. Based on her symptoms, biomarkers and imaging findings, a clinical diagnosis of myopericarditis was made. Treatment with colchicine, bisoprolol, furosemide and spironolactone were introduced. We observed a fast normalization of CK and troponin T levels and clinical improvement. The RT-PCR tests performed yielded negative results for dengue, chikungunya, cytomegalovirus, Epstein Barr virus, herpesvirus, varicella zoster virus, parvovirus B19 and enteroviruses. Serologies were negative for human immunodeficiency virus (HIV), hepatitis B (HBV) and C (HCV) virus. Five months later, the left ventricular ejection fraction was 74% and revealed a minimum pericardial effusion, without signs of cardiac tamponade. After one year, the cMRI showed absence of pericardial effusion (Fig. 1C) and a small area of intramyocardial hyperintensity involving the basal medial segment of the anterior septal wall. This was compatible with a fibrosis with characteristic pattern non-ischemic cardiomyopathies (Fig. 1D). The patient presented significant clinical improvement.

ZIKV, dengue and chikungunya detection was performed by rRT-PCR employing the commercially available kit ZDC from *Instituto de Tecnologia em Imunobiológicos Biomanguinhos*.. Serum sample was screened for five herpesviruses: herpes simplex virus type 1and 2 (HSV-1 and HSV-2), Cytomegalovirus (CMV), varicella zoster virus (VZV), Epstein-Barr virus (EBV) by multiplex PCR [12]. Seminested PCR was used for the identification of Parvovirus B19 [13] and of all enteroviruses [14]. Serology for HIV, HBV and HCV were assessed by immunochromatographic rapid tests.

The patient was enrolled in a prospective cohort study designed to assess the persistence of ZIKV in different body fluids. The study protocol was approved by institutional ethics committee as described by Calvet et al., 2018 [15]. Written informed consent was obtained from the patient.

## Discussion and conclusions

We describe a ZIKV infection case presenting with rarely described myopericarditis. Viral infections are among the most common reported causes of myopericarditis in high-income countries especially the genus enteroviruses, herpesviruses, adenoviruses, influenza A and B, parvovirus B19, HBV, HCV, HIV, and Varicella [16]. Potential infectious causes of myopericarditis were ruled out in this case. Immunopathic causes of myopericarditis are vasculitis in connective tissue diseases, inflammatory bowel diseases, radiation-induced and drug-induced myopericarditis [16]. Congenital ZIKAV syndrome has been associated with neurological disorders. Involvement of other organs such as the heart has also been reported, but without clinical and complementary exam evidence of myocarditis or pericarditis. A previous study reported that 13.5% of the echocardiograms performed in children with congenital ZIKV infection presented findings compatible with congenital heart disease [5]. The most common echocardiographic findings were ostium secundum atrial septal defect and ventricular septal defect [5]. Chan et al. observed the presence of viral RNA in the cardiac muscle of mice infected by ZIKV [6]. Acute inflammatory disease of the myocardium or pericardium has been rarely described in ZIKV infection. Aletti et al*.* documented transitory myocarditis associated with the ZIKV. The diagnosis of cardiac involvement was made by the increase of CPK, troponin T and ST-segment elevation in anteroseptal region, associated with serological confirmation of ZIKV infection. The cMRI performed 10 days later showed a slight left ventricular dilatation [7]. The evaluation of critical patients, with unfavorable outcome, by Zonneveld et al., revealed only elevation of CK and its CK-MB fraction, without electrocardiographic changes suggesting acute myocardial infarction [8]. Carta et al. detected the presence of arrhythmias in patients with cardiac symptoms from ZIKV endemic area. The main manifestations at the ECG were acute atrial fibrillation, ventricular arrhythmias, and non-sustained atrial tachycardia. Five of the six heart failure patients had a low ejection fraction [9]. This study focused on the potential threat that ZIKV may pose to the heart like others arboviral diseases [10]. Villamil-Goméz et al. suggested that ZIKV can frequently affect the heart, as shown by electrocardiographic changes and pericardial effusion by echocardiogram, but without clinical manifestations of cardiac involvement. It is possible that the described changes could be part of a systemic inflammatory response rather than a direct viral aggression [11]. The cMRI showed fibrosis in the acute phase. Two other cases reported fibrosis in the acute phase of myopericarditis associated with ECHO virus and with primary HIV infection in young people [17, 18]. Although cRMI is mandatory to have a non-invasive confirmation of the clinical diagnosis in high-income countries, it is not highly accessible in low-income countries because of its high cost [19]. Our patient was managed with beta-blocker, diuretics and colchicine. Colchicine has been shown to be useful in myopericarditis due to its mechanisms of down, such as down regulation of multiple inflammatory pathways and modulation of inate immunity [20]. A previous study has shown that colchicine was associated with complete resolution of myocarditis in 63% of cases [21]. A recent report confirmed the benefit of colchicine in 86 patients with myopericarditis, with 64% of complete resolution on cardiac resonance at one year of follow-up. We did not use in this case non-steroidal anti-inflammatory because of the high risk of acute kidney injury in a patient with shock and rhabdomyolysis [22].

We report a rare case of viral myopericarditis likely caused by ZIKV infection. The case described was unusual because the virus-induced myopericarditis resolved quickly and without sequelae. However, the cRMI performed after one year revealed myocardial fibrosis. Knowing the possible cardiac impact of ZIKV, careful monitoring of its function and rhythm should be done in ZIKV infected patients who present with any cardiac symptoms.

## Data Availability

Data sharing is not applicable to this case report as no datasets were generated or analysed during the current study.

## Abbreviations

ZIKV: Zika virus
FMT-HVD: *Tropical Medicine Foundation Doctor Heitor Vieira Dourado*
rRT-PCR: Real time reverse transcriptase polymerase chain reaction
CK: Creatine kinase
CK-MB: Creatine kinase-muscle/brain
CKMB: Creatine kinase-muscle mass
ECG: Electrocardiogram
echo: Echocardiogram
cMRI: Cardiac magnetic resonance imaging
HIV: Human immunodeficiency virus
HBV: Hepatitis B virus
HCV: Hepatitis C virus
HSV-1: Herpes simplex virus type 1
HSV-2: Herpes simplex virus type 2
CMV: Cytomegalovirus
VZV: Varicella zoster virus
EBV: Epstein–Barr virus
